# Graphene-Polymer Composites

**DOI:** 10.3390/polym13050685

**Published:** 2021-02-25

**Authors:** Artur M. Pinto, Fernão D. Magalhães

**Affiliations:** LEPABE—Laboratory for Process Engineering, Environment, Biotechnology and Energy, Faculty of Engineering, University of Porto, 4200-465 Porto, Portugal; arturp@i3s.up.pt

Graphene-polymer nanocomposites continue to gain interest in diverse scientific and technological fields. Graphene-based nanomaterials present the advantages of other carbon nanofillers, like electrical and thermal conductivity, while having significantly lower production costs when compared to materials such as carbon nanotubes, for instance. In addition, in the oxidized forms of graphene, the large specific area combined with a large quantity of functionalizable chemical groups available for physical and/or chemical interaction with polymers, allow for good dispersion and tunable binding with the surrounding matrix. Other features are noteworthy in graphene-based nanomaterials, like their generally good biocompatibility and the ability to absorb NIR radiation, allowing for the use in biomedical applications, such as drug delivery and photothermal therapy.

This Special Issue provides an encompassing view on the state of the art of graphene-polymer composites, showing how current research is dealing with new and exciting challenges. The published papers cover topics ranging from novel production methods and insights on mechanisms of mechanical reinforcement of composites, to applications as diverse as automotive and aeronautics, cancer treatment, anticorrosive coatings, thermally conductive fabrics and foams, and oil-adsorbent aerogels. The most impactful works are summarized in this editorial.

Taking advantage of graphene’s unique mechanical properties, Irez et al. newly designed lightweight and cost-efficient composite materials for the aeronautic industry using recycled fresh scrap rubber, epoxy resin, and graphene nanoplatelets (GnPs). After manufacturing the composites, their bending strength and fracture characteristics were investigated by three-point bending (3PB) tests. Halpin–Tsai homogenization adapted to composites containing GnPs was used to estimate the moduli of the composites, and satisfactory agreement with the 3PB test results was observed. In addition, 3PB tests were simulated by the finite element method incorporating the Halpin–Tsai homogenization, and the resulting stress–strain curves were compared with the experimental results. Mechanical test results showed that the reinforcement with GnPs generally increased the modulus of elasticity as well as the fracture toughness of these novel composites. The typical toughening mechanisms observed were crack deflection and cavity formation. Considering the advantageous effects of GnPs on these novel composites and the cost efficiency gained by the use of recycled rubber, these composites have the potential to be used to manufacture various components in the automotive and aeronautic industries as well as smart building materials in civil engineering applications [1]. The mechanical behavior of graphene/polymer interfaces in the graphene-reinforced epoxy nanocomposite is one of the factors that dictates the deformation and damage response of the nanocomposites. Koloor et al. performed hybrid molecular dynamic (MD) and finite element (FE) simulations of a graphene/polymer nanocomposite to characterize the elastic-damage behavior of graphene/polymer interfaces under a tensile separation condition. The MD results show that the graphene/epoxy interface behaves in the form of an elastic-softening exponential regressive law. The FE results verified the adequacy of the cohesive zone model in accurate prediction of the interface damage behavior. The graphene/epoxy cohesive interface was characterized by normal stiffness, tensile strength, and fracture energy of 5 × 10^−8^ (aPa nm^−1^), 9.75 × 10^−10^ (nm), 2.1 × 10^−10^ (N nm^−1^), respectively, followed by an exponential regressive law with the exponent, α = 7.74. It was shown that the commonly assumed bilinear softening law of the cohesive interface could lead up to a 55% error in the predicted separation of the interface [2].

Using a different resin, Liu et al. prepared graphene-reinforced tung oil (TO)-based unsaturated polyester nanocomposites via in situ melt polycondensation integrated with Diels–Alder addition. Functionalized graphene sheets derived from graphene oxide (GO) were then extracted from the obtained nanocomposites and carefully characterized. The mechanical and thermal properties of the TO-based unsaturated polyester resin (UPR) were greatly improved by the incorporation of GO. At the optimal GO content (0.10 wt.%) the nanocomposite showed a tensile strength and a tensile modulus of 43.2 MPa and 2.62 GPa, and *T_g_* of 105.2 °C, which were 159%, 191%, and 49.4% higher than those of the unreinforced UPR/TO resin, respectively. Compared to neat UPR, the biobased UPR nanocomposite with 0.10 wt.% of GO even demonstrated superior comprehensive properties (comparable stiffness and *T_g_*, while better toughness and thermal stability). Therefore, the developed biobased UPR nanocomposites are very promising to be applied in structural plastics [3].

Functional fabrics have gained attention as an environmentally-friendly synthesis route. Yang et al. developed novel bamboo pulp fabrics with thermal conductivity properties by coating the fabric with graphene and cellulose nanocrystal (G/CNC) solutions. The influences of G and CNC concentrations on the properties of fabrics were studied. It was found that the viscosities of the G/CNC solutions increased with an increase of G contents. G had an obvious thickening effect. Furthermore, compounded fabrics with different G and CNC contents (GCBPFs) were prepared and extensively characterized in terms of their thermal and mechanical properties as well as morphology. The ultimate thermal conductivity, bursting strength, and tensile strength of the GCBPF were 0.136 W/m K, 1.514 MPa, and 25.8 MPa, with 4 wt.% CNC and 3 wt.% G contents. The results demonstrated that the as-fabricated GCBPFs with favorable thermal conductivity could be applied as a novel fast cooling textile for the clothing industry [4].

Under the current situation of frequent oil spills, the development of green and recyclable high-efficiency oil-absorbing aerogel materials has attracted wide attention from researchers. Zhou et al. reported a high-strength, three-dimensional hydrophobic cellulose nanofiber (CNF)/polyvinyl alcohol (PVA)/graphene oxide (GO) composite aerogel with an anisotropic porous structure, which was fabricated by directional freeze-drying technology using anisotropically grown ice crystals as a template, followed by hydrophobic treatment with a simple dip-coating process. The prepared composite aerogel presented anisotropic multi-level pore microstructures, low density (17.95 mg/cm^3^), high porosity (98.8 %), good hydrophobicity (water contact angle of 142°), and great adsorption capacity (oil absorption reaching 96 times its own weight). More importantly, the oriented aerogel had high strength, whose compressive stress at 80% strain reached 0.22 MPa, and could bear more than 22 times its own weight without deformation. Therefore, a CNF/PVA/GO composite aerogel could be prepared by a simple and easy-to-operate directional freeze-drying method, being a promising absorbent for oil-water separation [5].

Besides the graphene-based materials intrinsic properties and the interactions with the polymer matrix, the amounts of filler used and the composite production methods can have an impact on its final properties. Huang et al. studied the effects of increasing graphene nanosheet (GNS) concentrations on variations in the structure and properties of electrospun GNS-filled poly(trimethylene terephthalate) (PTT/GNS) composite fibers, such as in its morphology, crystallization behavior, mechanical properties, and electrical conductivity. The effects of GNS addition on solution rheology and conductivity were also investigated. GNSs were embedded in the fibers and formed protrusions. The PTT cold crystallization rate of PTT/GNS composite fibers increased with the gradual addition of GNSs. A PTT mesomorphic phase was formed during electrospinning, and GNS could induce the PTT mesomorphic phase significantly during PTT/GNS composite fiber electrospinning. The PTT/GNS composite fiber mats (CFMs) became ductile with the addition of GNS. The elastic recoveries of the PTT/GNS CFMs with 170 °C annealing were better than those of the as-spun PTT/GNS CFMs. Percolation scaling laws were applied to the magnitude of conductivity to reveal the percolation network of electrospun PTT/GNS CFMs. The electrical conductivity mechanism of the PTT/GNS CFMs differed from that of PTT/GNS composite films. Results showed that the porous structure of the PTT CFMs influenced the performance of the mats in terms of electrical conductivity [6]. Hendrix et al. developed a method exploiting high-speed elongational flow in a novel designed batch mixer, creating a distribution of pristine few to many layer graphene flakes. The method focuses on exfoliating in a molten polyamide 66 (PA66) matrix, creating a graphene reinforced polymer matrix composite (G-PMC). The process revealed that high-speed elongational flow was able to create few layer graphene. Graphite exfoliation was found driven in part by diffusion, leading to the intercalation of PA66 in graphite. The intercalated structure lead to increases in the hydrogen bonding domain, creating anisotropic crystal domains. The thermal stability of the G-PMC was found to be dependent on the degree of exfoliation, the PA66 crystal structure, and composite morphology. The aim of this research was to characterize uniquely produced graphene containing polymer matrix composites using a newly created elongational flow field. Using elongational flow, graphite can be directly exfoliated into graphene within a molten polymer [7].

Graphene impermeability against water, oxygen, and ions diffusion, together with its large surface area, make it an outstanding material for application in anticorrosion surface coatings. Lei et al. studied the corrosion behavior of zinc-rich epoxy primers or paints (ZRPs) with different conducting polyaniline-grafted graphene (PANI/Gr) contents. The conductivity of the formed PANI/Gr nanosheets was significantly improved by employing Gr as the inner template to synthesize the PANI. The protective properties and the electrochemical behavior of coatings with artificial defects were investigated by monitoring the free corrosion potential versus time and by using localized electrochemical impedance spectroscopy (LEIS). A synergetic enhancement of the physical barrier role of the coating and the zinc sacrificial cathodic protection was achieved in the case of ZRP including PANI/Gr nanosheets. In addition, the ZRP mixed with the PANI/Gr at a content of 0.6% exhibited the best anticorrosion performance across the range of investigated PANI/Gr contents [8].

Graphene and graphene polymer composites have been widely explored for the development of medical implants, antibacterial materials, biosensors, drug and gene delivery systems, as well as phototherapy platforms. Gurunathan et al. produced graphene oxide–green platinum nanoparticles (GO-PtNPs) using vanillin and studied its effect on human prostate cancer cells (LNCaP). GO-PtNP cytotoxicity in LNCaP cells was demonstrated by measuring cell viability and proliferation. Both decreased in a dose-dependent manner compared to that by GO or PtNPs alone. GO-PtNP cytotoxicity was confirmed by increased lactate dehydrogenase release and membrane integrity loss. Oxidative stress induced by GO-PtNPs increased malondialdehyde, nitric oxide, and protein carbonyl contents. The effective reactive oxygen species generation impaired the cellular redox balance and eventually impaired mitochondria by decreasing the membrane potential and ATP level. The cytotoxicity to LNCaP cells was correlated with increased expression of proapoptotic genes (p53, p21, Bax, Bak, caspase 9, and caspase 3) and decreased levels of antiapoptotic genes (Bcl2 and Bcl-xl). Activation of the key regulators p53 and p21 inhibited the cyclin-dependent kinases Cdk2 and Cdk4, suggesting that p53 and p21 activation in GO-PtNP-treated cells caused genotoxic stress and apoptosis. The increased expression of genes involved in cell cycle arrest and DNA damage and repair as well as the increased levels of 8-oxo-deoxyguanosine and 8-oxoguanine suggested that GO-PtNPs potentially induce oxidative damage to DNA. Thus, GO-PtNPs are both cytotoxic and genotoxic. LNCaP cells appear to be more susceptible to GO-PtNPs than to GO or PtNPs. Thus, GO-PtNPs have potential as an alternate and effective cancer therapeutic agent. In other words, this work shows that the combination of graphene oxide with platinum nanoparticles opens up new perspectives in cancer therapy. However, further detailed mechanistic studies are required to elucidate the molecular mechanism of GO-PtNPs induced cytotoxicity in prostate cancer [9]. Costa-Almeida et al. used a one-step thermal reduction and noncovalent chemical functionalization process to produce PEGylated reduced nanographene oxide (rGOn-PEG) from nanographene oxide (GOn). Nanomaterials were characterized in terms of particle size, dispersion stability, chemistry, and photothermal properties, in view of its use for photothermal therapy (PTT) of nonmelanoma skin cancer. GOn infrared spectrum presented more intense bands assigned to oxygen containing functional groups than observed for rGOn-PEG. GOn C/O ratio decreased more than 50% comparing with rGOn-PEG and nitrogen was present in the latter (N at.% = 20.6) due to introduction of PEG-NH_2_. Thermogravimetric analysis allowed for estimating the amount of PEG in rGOn-PEG to be of about 56.1%. Simultaneous reduction and PEGylation increased the lateral dimensions from 287 ± 139 nm to 521 ± 397 nm, as observed by transmission electron microscopy and dynamic light scattering. It was found that rGOn-PEG exhibited roughly 13-fold higher absorbance in the near-infrared radiation (NIR) region, as compared to unmodified GOn. Low power (150 mW cm^−2^) NIR irradiation using LEDs resulted in rGOn-PEG heating up to 47 °C, which is within the mild PTT temperature range. PEGylation strongly enhanced the dispersibility of rGOn in physiological media (phosphate buffered saline, fetal bovine serum, and cell culture medium) and also improved the biocompatibility of rGOn-PEG, in comparison to GOn (25–250 μg mL^−1^). After a single NIR LED irradiation treatment of 30 min, a decrease of ≈38% in A-431 cells viability was observed for rGOn-PEG (250 μg mL^−1^). Together, these results demonstrate the potential of irradiating rGOn-PEG using lower energy, cheaper, smaller, and safer LEDs as an alternative to high-power lasers for NIR mild hyperthermia therapy of cancer, namely nonmelanoma skin cancer [10].

## Data Availability

No new data were created or analyzed in this study. Data sharing is not applicable to this article.
